# Maxillary epithelioid hemangioendothelioma: an especially rare malignant tumor mimicking periodontal disease

**DOI:** 10.1186/s12903-020-01291-4

**Published:** 2020-11-06

**Authors:** Gintaras Januzis, Dovydas Sakalys, Martynas Mantas Krukis, Dmitrij Seinin

**Affiliations:** 1 Department of Maxillofacial Surgery, Faculty of Odontology, Lithuanian University of Health Sciences, A. Ramanausko – Vanago g. 8-4, Kaunas, Lithuania; 2 National Center of Pathology, Vilnius University Hospital Santaros Clinics, Vilnius, Lithuania

**Keywords:** Epithelioid hemangioendothelioma, Oral cancer, Vascular tumor, Endothelial cells, Periodontitis, Gingival pathologies, Immmunohistochemical markers, CD31, CD34, ERG

## Abstract

**Background:**

Epithelioid hemangioendothelioma (EHE) is an especially rare, low-grade malignant vascular tumor that, according to WHO classification, is described as locally aggressive tumor with possible metastasis and makes up 1% of all vascular tumors. EHE is characterized by the accumulation of round, eosinophil-infiltrated endothelium cells; with vacuolation of their cytoplasm; frequent angiocentric inflammation; and myxohyaline stroma. This tumor is usually found in the liver, lungs, and bones and is especially rare in the mouth.

**Case presentation:**

We present an 18-year-old Caucasian female whose oral cavity lesion had been misdiagnosed as marginal periodontitis. The patient was treated improperly for 2 years until she was referred to a maxillofacial surgeon. The patient complained only about gingival recession in the palatal area of her upper-right-side 13th, 14th, and 15th teeth. The lesion’s clinical appearance was of locally ulcerated painless lesion that affect the underlying bone as seen in X-rays in the palatal side of the right canine and the first and second premolars. Patient underwent surgery for her present defect and reconstruction using allogenic bone transplant. The diagnosis of EHE was based on the bony destruction as seen in x-rays and in the accumulation of tumor cells that were 100% positive to CD31; CD34 and ERG to endothelial markers. During the 31-month follow-up period, the patient exhibited no clinical and radiographic complications.

**Conclusions:**

With this clinical case, we demonstrate that this rare tumor must be included in differential diagnoses of periodontal pathologies to perform histomorphological examination in a timely manner, which could lead to correct diagnosis and adequate treatment.

## Background

The term hemangioendothelioma (HE) was first proposed by Borrmann in 1899 as a low-grade malignancy vascular tumor [1]. HEs can relapse but very rarely metastasize, significantly less often than angiosarcomas do [1]. According to their histopathological characteristics, hemangioendotheliomas are classified as kaposiform, Dabskos, or epithelioid. The kaposiform type is usually found in infants and in surface soft tissues, and is associated withcoagulopathy and thrombocytopenia (Kasabach–Merritt syndrome) [1, 2]. The Dabskos type is usually found in young people and mostly in limbs [1].

Epithelioid hemangioendothelioma (EHE) is an especially rare, low-grade malignant vascular tumor that, according to the WHO’s classification, is described as locally aggressive tumor with possible metastasis that makes up 1% of all vascular tumors; [3–6] The gender predilection varies, and the average age of EHE patients is 36 years [7]. EHE is histological characterized by the proliferation of round, eosinophil-infiltrated endothelium cells; the vacuolation of cytoplasm, frequent angiocentric inflammation, myxohyaline stroma [8]. The first documented case was reported in 1982. Among EHE cases, 21% are in the liver, 18% are in the liver and lungs, 12% are in the lungs, 14% are in bones, and rare in the mouth [3]. According to our literature review, only 38 EHE cases in the mouth have been published, and bone destruction in the maxilla manifested in only 3 of those cases [8, 10–23]. This tumor, its growth is slow, and its symptoms are similar to those of chronic inflammation; therefore, it is highly difficult to diagnose.

Our clinical case is unique in terms of its clinical course and the simulation of other periodontal diseases, and its presentation will help other' specialists to diagnose the lesion and adapt a treatment.

## Case presentation

**Fig. 1:**
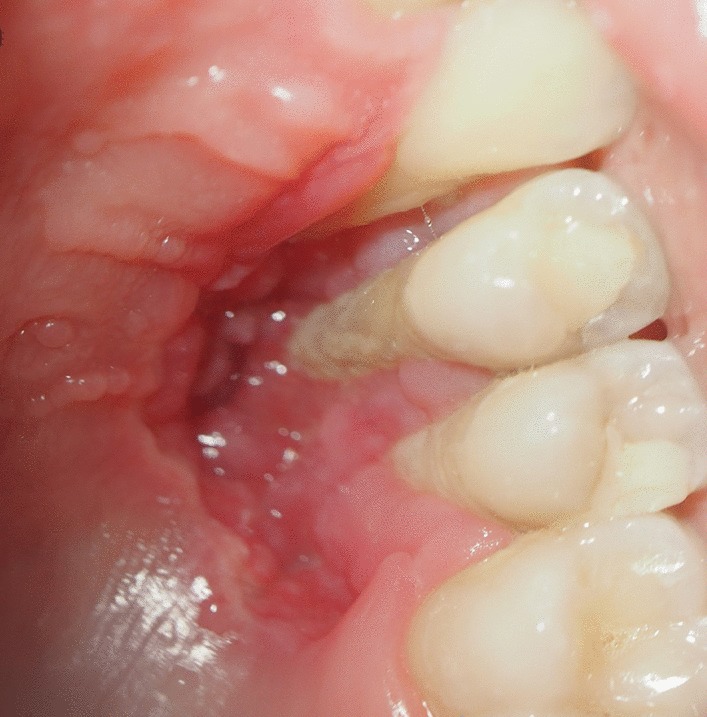
Clinical photograph of defect in maxilla

**Fig. 2.**
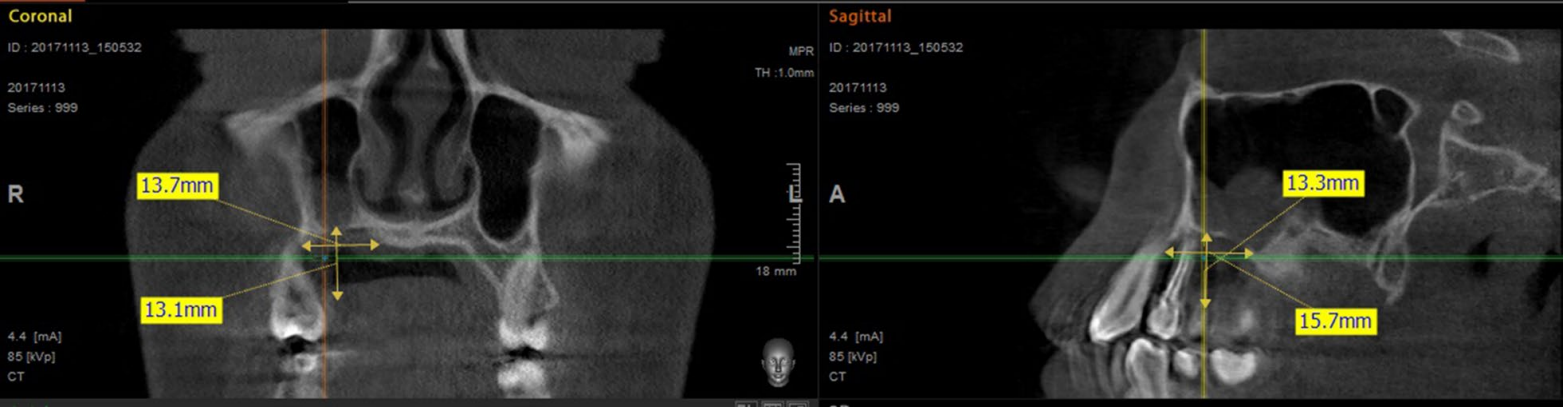
3D computed tomography showing bone defect at 13, 14, 15 teeth region

At 18 year old, a Caucasian female patient without any systemic diseases or drug use was presented for evaluation of gingival problems around her upper right premolar. Marginal periodontitis was diagnosed by her Dentist and the patient was referred to a Periodologist for root scaling. During the next two years, the patient was undergone perio-treatments under anti-inflammatory medications and had root canal treatment of her second premolar one year later. Biopsy of the lesion was not taken but the lesion was not gone but slowly progressed and therefore the patient was referred to a maxillofacial surgeon at our clinic. During her first consultation with the maxillofacial surgeon, the patient only complained about gingival recession in the palatal area of her upper-right-side teeth. The initial examination showed that the canine and both premolars had second-grade mobility (Fig. 1). The probing depth of teeth 13, 14, and 15 was < 3 mm on the buccal side and 5 mm at the palatal side as the palatal gingiva were recessed leaving exposed the fist premolar and canine and less the second premolar whose exposed root surface was covered with dental plaque. The patient’s jaw underwent 3D computed tomography, which revealed bone destruction in the defect area reaching the maxillary sinus, whose mucosa was locally thickened (Fig. 2). The condition of periodontium around other teeth was fine. The probing depth was < 3 mm around all other teeth. The chronic long-term lesion, with its unknown cause and unusual localization (defect in the palate side, while the marginal buccal surface bone was uninjured), caused confusion to the Oral Surgeon as the clinical findings were not characteristic of oncological tumors: such as the absence of induration of adjacent soft tissues, the boundaries were clearly visible, and the unpleasant smell characteristic of tissue collapse was not detected from the lesion. Moreover, no regional lymph nodes were palpable, and none of the patient’s close relatives were having similar lesions.

It was decided to remove all three teeth (13th, 14th and 15th), perform a removal of the altered soft tissues, and evaluate them histologically. After radical surgery of the defect, it was decided to reconstruct the area using allogenic bone transplant. Surgery was performed under local anesthesia. The initial prosthetic treatment plan was to insert dental implants in the area of teeth 13 and 15 about 6 months after the excision of the altered tissues and to make a fixed 3-tooth bridge on the dental implants afterward. However, after the diagnosis was histologically confirmed, prosthetic treatment was delayed for 12 months after excision in case relapse did not occur.

**Fig. 3:**
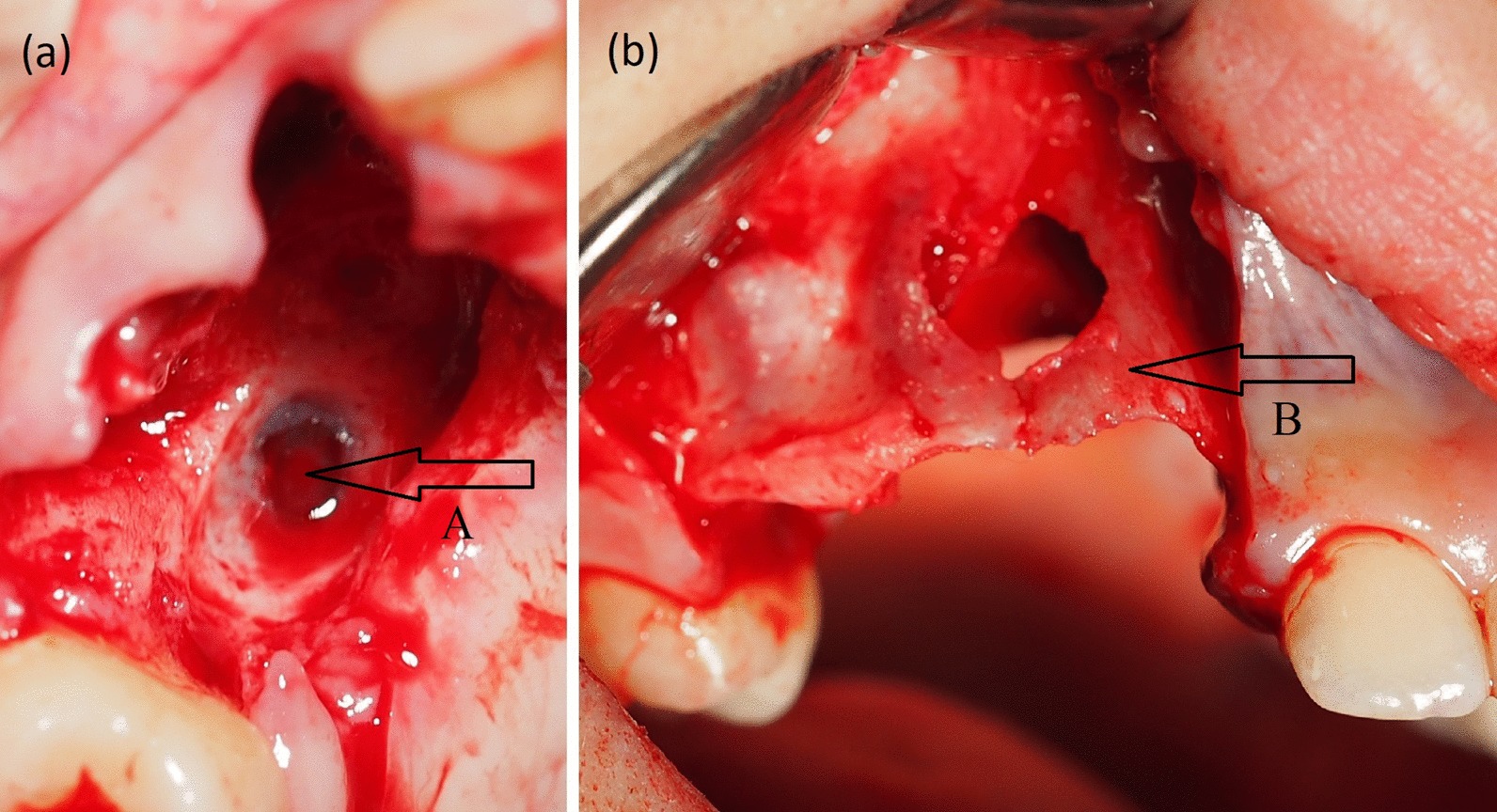
**a**, **b** Clinical photographs after excision of tumor. **a** Intact Schneiderian membrane, **b** remained buccal wall of the alveolar ridge

The teeth were removed during surgery, and the altered soft tissues were removed based on the clinical view, within the boundaries of healthy tissues. Soft tissues with granulations and teeth were easily separated from the bone. The bone relief was uneven but hard and was covered in a compact bone layer. The formed bone defect was more similar to uneven bone lysis than to destruction (Fig. 3a, b). All of the resected tissues were sent for histomorphological evaluation. After the teeth with altered tissues were removed, the bone window (uzura) to the sinus was visible, but the integrity of the membrane was intact. The membrane, as visible through the window, looked unchanged.

Soft tissue deficit was present from the crest to the hard palate. Tumor-free margins were about 20 mm wide mesiodistally and about 15 mm wide mediolaterally (Fig. 3a, b). Sticky bone graft with platelet concentrate made from venous blood according to the PRGF Endoret® method, in combination with demineralized freeze-dried bone allograft was chosen for restoration of bone defect [9]. The soft tissue defect was covered with free gingival flap from the buccal to the palatal side. Antibiotic therapy (875 mg of amoxicillin with 125 mg clavulanic acid twice a day for 7 days) and painkillers (25 mg of Dexketoprofen according to the patient’s needs, no more than 3 times per day for 5 days) were prescribed after surgery. The wound healed without complications, and the sutures were removed after 10 days.

**Fig. 4:**
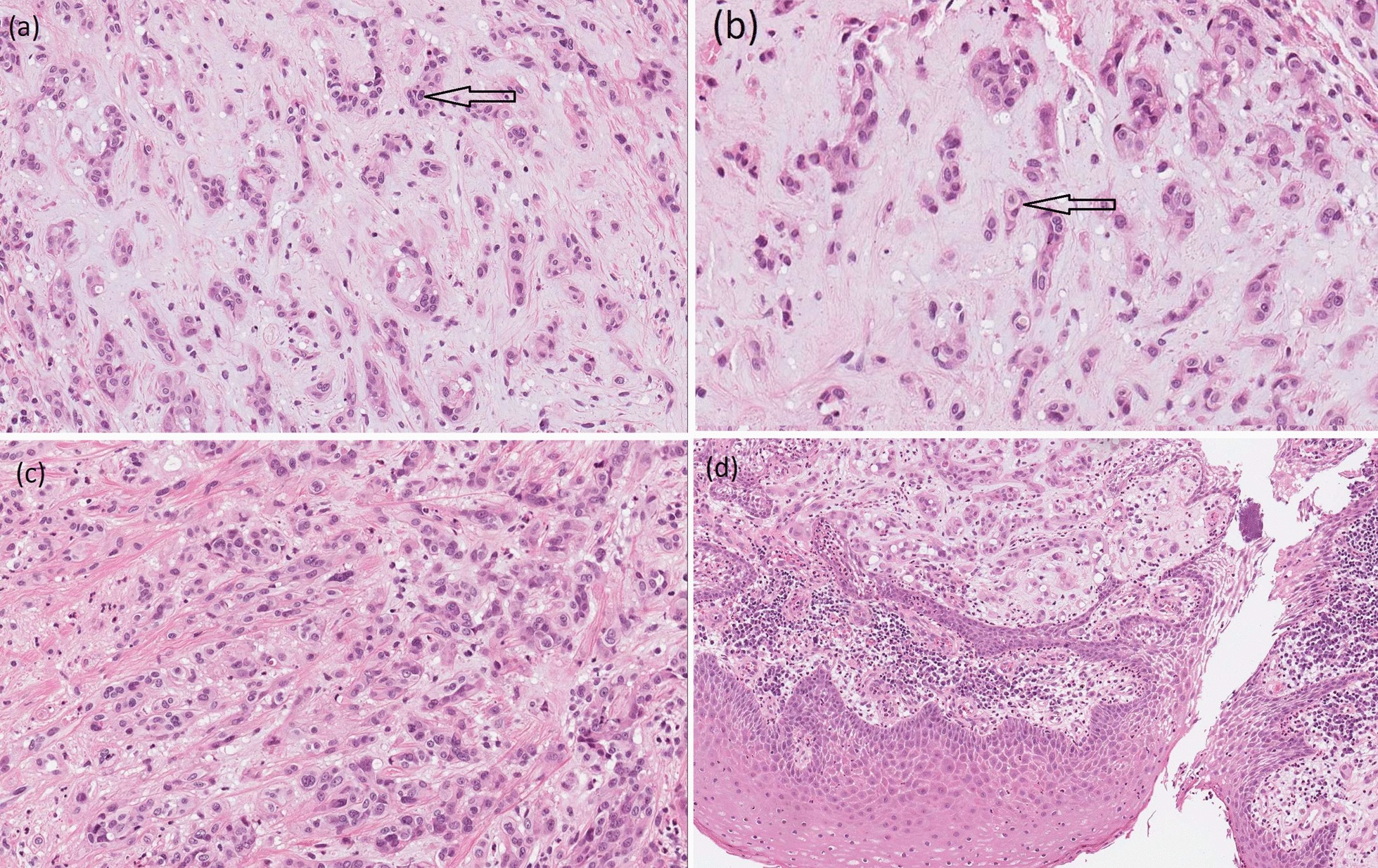
**a**–**d** Atypical epithelial cells (arrows) of different shapes in fibromycoidic stroma with trabecular structures (**a**) × 20 and (**b**); atypical epithelial cells with eosinophilic cytoplasm and insignificant polymorphic oval nuclei (**c**) × 20; tumor gingival mucosa with normal structure of stratified squamous cell epithelium (**d**) × 10 (haematoxylin and eosin staining)

**Fig. 5:**
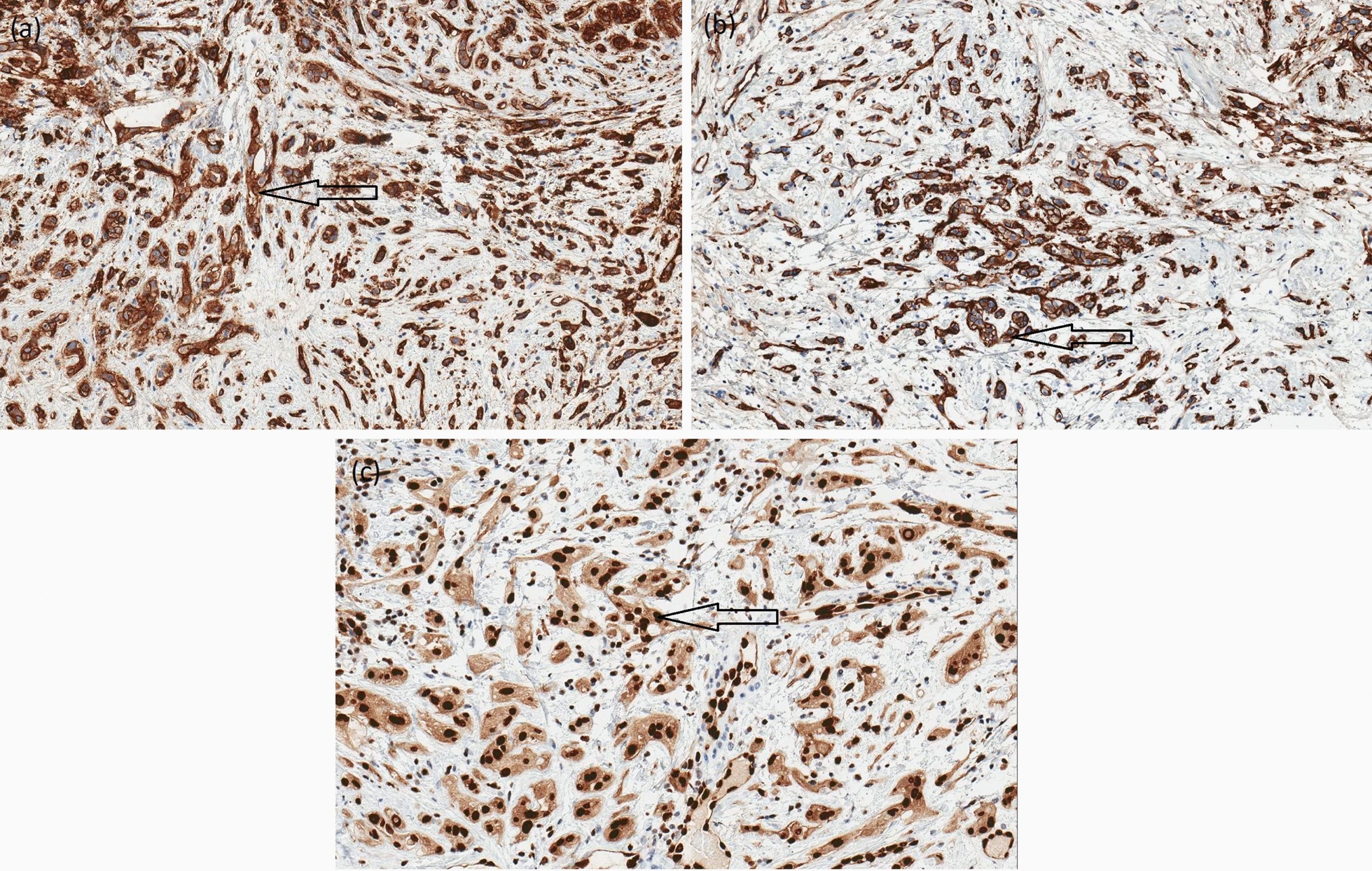
**a**–**c** Immunohistochemical staining: 100% Positive tumor cells cytoplasmic reaction (arrow) for CD31 × 10 (**a**); 100% positive tumor cells cytoplasmic reaction (arrow) for CD34 × 10 (**b**); 100% positive tumor cells nuclei reaction (arrow) for ERG × 20 (**c**)

The excised gingivae and adjacent oral mucosa sent for histological examination were partially ulcerated while the underlying submucosa was consisted of complexes of atypical epithelial cells of different shapes in fibromycoidic stroma (Fig. 4a–d). These cells had eosinophilic cytoplasm, insignificantly polymorphic oval cores, and isolated mitoses. Part of the cytoplasm of the atypical cells contained vacuoles with erythrocytes in openings. Immunohistochemical staining with endothelial markers CD31, CD34, and ERG was positive for 100% of the tumor cells (Fig. 5a–c), and reaction with the epithelial marker PanCK was positive for 10% of the tumor cells. The proliferative activity of Ki-67 was about 5%. ''The included bone did not reveal any infiltration from tumor cells. The final histomorphological conclusion was epithelioid hemangioendothelioma pT1b of low malignancy in the periodontal tissues and palatal mucous membrane.

The further examination of patient after histopathtological results was set up during the consultation with oncologist, who indicated the need of thoracic X-ray and abdominal ultrasound to identify any possible distant metastasis. An ultrasound head and neck region examination revealed isolated II A group neck lymph nodes up to 0.5 cm in diameter, which were considered to be reactive. Suspicious lymph nodes were not found. Ultrasound of the abdomen and chest X-ray did not show any pathology.

**Fig. 6:**
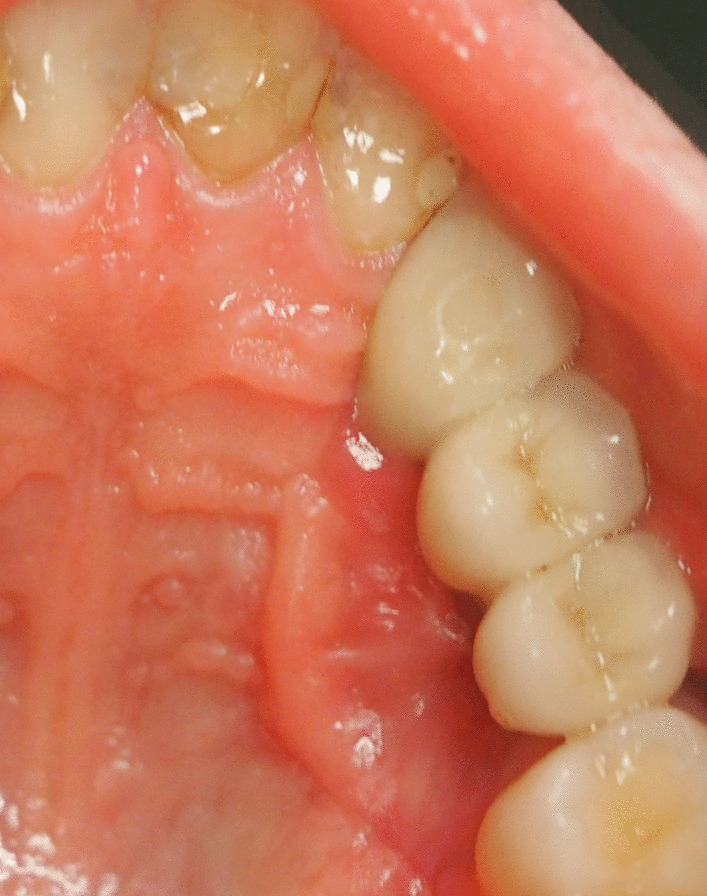
Clinical photograph taken 31 months after excision and 12 months after prosthetic

After 31 months, there have been no clinical signs of relapse. Three-dimensional computed tomography of the jaws was carried out 5 months after surgery and showed that the augmented bone had retained its shape but without complete mineralization. Considering possible relapse, the patient was scheduled for additional consultations and examinations after 3, 6, and 12 months. Prosthetic treatment was started 12 months after the tumor’s excision. Two dental implants were inserted in the area of the 13th and 15th teeth. Six months after implantation, prosthetic implants were installed with a 3-unit zirconium ceramic bridge (Fig. 6). The patient had no complaints about her condition 12 months after this prosthetic treatment.

## Discussion and conclusions

According to a data review, only 38 cases of EHE in the mouth cavity have been documented. [8, 10–23]. EHE was found in the gingivae of mandible in 13 cases; in the gingiva of the maxilla in 12 cases; in the tongue in 7 cases; in the buccal mucosa, palate, lower mouth, and lips in 4 cases; in the mandible bone in 2 cases. The uniqueness of the presented case is indicated by the fact that only 13 cases including bone destruction have been documented, including only 3 cases in the maxilla [8, 10–16]. A retrospective evaluation of our described clinical case indicated that bone destruction appeared because of the extended lesion, which was caused by the late diagnosis.

According to the literature, many EHE patients do not experience symptoms, and only a very small proportion felt pain in the area of the tumor. Clinically, EHE in mouth manifests itself with nonspecific symptoms and usually appears as a benign nonpainful formation, although some cases included ulceration of the mucosa and signs of bone destruction. In the described cases, EHE was misdiagnosed as periodontitis, gingivitis, fibroma, papilloma, hemangioma, or even carcinoma [8, 10–23]. Epithelioid hemangioendothelioma should be differentiated from other mouth mucosa inflammations by the locality of its defect, its possible manifestation on only one surface of the alveolar process, and it not necessarily being accompanied by the pathologic mobility of teeth. In differentiating EHE from inflammation (gingivitis or periodontitis), a clear cause of inflammation such as bad hygiene, plaque, concrements, or traumatic occlusion is rarely found. Accurate and final diagnosis can only occur after histological examination. Histological EHE differential diagnosis with nonvascular tumors (such as carcinomas, melanomas, embryonal rhabdomyosarcomas, and epithelioid angiosarcomas) is based on data from an immunohistochemical examination: EHE cells are stained using endothelial markers CD31, CD34, and ERG, which are specific to vascular tumors. The endothelial markers in carcinomas, melanomas, embryonal rhabdomyosarcomas, and epithelioid sarcomas would be negative. The vimentin sign will also be negative for carcinomas. In the case of hemangiopericytoma, tumor cells will react positively with SMA. EHEs are different from hemangiomas and hemangioendotheliomas due to the former’s specific histologic and cytologic structure. As opposed to EHE, epithelioid angiosarcoma is a highly malignant vascular tumor with expressed polymorphism of atypical epithelioid cells and mitotic and proliferative Ki-67 activity. Because of its tendency to reappear after primary removal, literature sources suggest treating EHE by performing wide excisions in the boundaries of healthy tissues and regularly monitoring the patient after surgery [11]. In addition, the patient’s regional lymph nodes, lungs, and liver should be examined for possible metastases. Relapses were described in 10 cases, and only in 1 case was metastasis found in regional neck lymph nodes, 4 years after the tumor was excised [10]. No cases have been described of a patient death caused by EHE in the mouth.

The initial incisional biopsy in this case was not planned because the defect had inflammatorily altered tissues with little granulations; had no malignant looking tissues; had no endophytic or exophytic malignant tissue infiltration, either clinically or radiologically; and did not look malignant. Moreover, because of the highly damaged periodontal tissue of teeth 13, 14, and 15, we considered them to be indicated for extraction—this is another reason why we decided to solve the case with radical surgery.

The greatest limitation to our approach in this case was that the clinical presentation did not raise instant suspicion of a neoplastic lesion, so an initial biopsy was not performed. Another limitation was that defect was reconstructed by using an allogenic bone transplant in combination with plasma rich in growth factors, yet hyperexpression of some of the growth factors released by platelets is linked with oncological processes [24]. The clinical findings were not characteristic of oncological disease: there was no induration of adjacent soft tissues, the boundaries were clearly visible, and the unpleasant smell characteristic of tissue collapse was not present too at the damaged bone area. Moreover, no regional lymph nodes were enlarged. That was the main reasons why we decided to instantly reconstruct the defect with an allogenic bone graft mixed with plasma rich in growth factors and accelerate the patient’s recovery. However, no relapse has been observed after more than 20 months. The radical excision of the affected tissues ensured minimal risk of a possible relapse.

This clinical case demonstrates that diseases that appear to be gingivitis or periodontitis at first glance can have atypical chronic course, which is hard to identify according to the clinical symptoms. Moreover, such a rare occurrence of this hardly distinguishable tumor can easily mislead any clinician. One of the most important diagnostic tools for differential diagnosis is cytological and histological examination of pathologic lesions. Any results of such examinations will support or deny the diagnosis and support the assigned treatment in unusual cases. With this clinical case, we can demonstrate the long-term course of this chronic disease, which caused the patient to lose 3 teeth because of the lesion’s expansion. All of this could have been prevented by timely performed histomorphological examination, which could have led to correct diagnosis and adequate treatment. Our case shows the importance of clinicians to become familiar with this rare tumor.

## Data Availability

The data sets generated and analysed during the current study are not publicly available due to patient’s individual privacy may be compromised but are available from the corresponding author on reasonable request.

## Abbreviations

EHE: Epithelioid hemangioendothelioma
HE: Hemangioendothelioma
SMA: Smooth muscle actin
